# Cross-cultural Analysis of Models of the Relationship between the Cognitive Abilities and Academic Achievement in Primary School Education

**DOI:** 10.11621/pir.2021.0407

**Published:** 2021-09-25

**Authors:** Tatiana N. Tikhomirova, Artem S. Malykh, Irina A. Lysenkova, Sergey B. Malykh

**Affiliations:** a Lomonosov Moscow State University, Moscow, Russia; b Psychological Institute of Russian Academy of Education, Moscow, Russia; c Kyrgyz-Russian Slavic University, Bishkek, Kyrgyzstan

**Keywords:** Cross-cultural study, information processing speed, nonverbal intelligence, visuospatial working memory, number sense, academic achievement, primary school education, structural equation modeling

## Abstract

**Background:**

The cognitive predictors of academic achievement are associated both with basic cognitive abilities such as the information processing speed, number sense and visuospatial working memory, as well as with general ability including nonverbal intelligence. However, the ratio between cognitive development and school achievement can depend on sociocultural conditions.

**Objective:**

The results of a cross-cultural analysis of the relationship between cognitive development and academic achievement during primary education are presented. The analysis was conducted sampling schoolchildren from Russia and Kyrgyzstan, two countries that have a similar organization of the national education system but differ in the level of socioeconomic development.

**Design:**

The study involved 732 schoolchildren aged 7.7 to 11.8 years studying in Russia and Kyrgyzstan. Information processing speed, visuospatial working memory, and number sense were assessed using each of “Choice Reaction Time,” “Corsi Block-Tapping Test,” and “Number Sense” computerized tests.

**Results:**

According to the results, empirical data in both samples show that a model where in information processing speed signifies basic cognitive ability is a key predictor of nonverbal intelligence, working memory, and number sense, and each of these may contribute to individual differences in academic achievement. Notwithstanding the universality of this model, cross-cultural differences were seen to engender a reduction of schoolchildren’s academic achievements, given possible impacts of less favorable educational conditions.

**Conclusion:**

In the relationship between cognitive abilities and academic success at the primary school education, there are both similarities and differences between schoolchildren studying in Russia and Kyrgyzstan.

## Introduction

The search for psychological predictors of individual differences in the academic achievements of schoolchildren is a socially significant task and is associated with problem of the relationship between cognitive development and learning success.

According to previous studies and their meta-analyses, among the individual psychological characteristics that affect school success, the leading factor is attributed to the cognitive sphere (Peng, Kievit, 2020; Tikhomirova, Malykh, Malykh, 2020; Rohde, Thompson, 2007; Laidra, Pullmann, Allik, 2007; Luo, Thompson, Detterman, 2006). These report that cognitive development indicators can explain up to 60% of the variability in academic success (Falch, Sangren Massih, 2011; Luo et al., 2006). In this context, we analyze the following basic cognitive development indicators: information processing speed (the ability to accurately and quickly respond to stimuli); visuospatial working memory (the ability to retain small pieces of information about the shape of stimuli and their spatial localization); number sense (the accuracy of the perception and assessment of objects without counting); and general abilities such as nonverbal intelligence. These basic and general cognitive abilities can improve or, conversely, worsen the prospects and level of academic success during schooling (Tikhomirova et al., 2020). Additionally, it was shown that of all cognitive indicators, working memory has a special role in individual differences in learning, and is a significant predictor not only of current educational achievements, but also of successful learning in the future (Demetriou, Kazali, Kazi, Spanousis, 2020; Tikhomirova, 2017). Studies show on the one hand that information processing speed is directly related to academic performance, but on the other that these do not necessarily have a direct effect on academic success (Ti khomirova, Kuzmina, Malykh, 2020; Tikhomirova et al., 2020; Brown, Brockmole, Gow, Deary, 2012). The influence of number sense on the academic achievement of schoolchildren, including success in mathematics, depends on the specific aspect of this cognitive ability associated with symbolic or non-symbolic representations of quantity (Kuzmina, Tikhomirova, Lysenkova, Malykh, 2020; Gebius, Reynvoet, 2012; Halberda, Mazzocco, Fergenson, 2008).

The role of cognitive abilities can vary significantly depending on the criteria used to evaluate learning success (for example, teacher assessments or standardized state examinations, Tikhomirova, 2017), the level of schooling (primary school or high school, Tikhomirova et al., 2020), academic discipline (language or mathematics, Tosto et el., 2019), etc. Previous studies have also reported on the mutual influence of cognitive development indicators and the specifics of their impact on academic success during schooling. For example, information processing speed, visuospatial working memory and intelligence fully explain heightened number sense associated with the operations of non-symbolically expressed quantities during primary school education (Kuzmina et al., 2020). In addition, intelligence is a moderator of the connection between various aspects of number sense that affect academic performance in mathematics but also changes with the influence of the first two years of primary education (Kuzmina et al., 2020; Tikhomirova et al., 2020). These data make it necessary to study the joint influence of cognitive abilities on academic success in different stages of schooling, e.g. primary, secondary or high school education. In general, it has been demonstrated that at different levels of schooling, the influences of cognitive abilities have registered differences in school performance (Tikhomirova et al., 2020; Demetriou, Markis, Tachmatzidis, Kazi, Spanousis, 2019). Most significantly, working memory affects school achievement, particularly during the period of primary school education between the ages of 9 and 13 (Demetriou et al., 2019).

In studies conducted using the two-factor model of intelligence as a framework, statistical arguments were presented to demonstrate the influence of cognitive indicators on academic success through general cognitive ability, “g” (Kranzler, Benson, Floyd, 2015). These studies assumed that the cognitive indicators are loaded on the factor “g” to varying degrees, which affects the efficiency of assimilation of new knowledge and skills during the schooling process.

A number of studies have, however, demonstrated an opposite contribution of cognitive indicators to academic achievements of schoolchildren (Geary, 2011; Rindermann, Flores-Mendoza, Mansur-Alves, 2010; Rohde, Thompson, 2007), underlining the importance of overall academic success, generally evaluated on the basis of several indicators such as grades in several school subjects or several types of assessment in one subject (Tikhomirova et al., 2020). Other studies have noted high correlation coefficients between general cognitive ability and general academic success ranging from 0.77 to 0.94 (Kaufman, Reynolds, Liu, Kaufman, McGrew, 2012).

These works have provided data on more complex models of the relationship between the cognitive sphere and academic success (Tikhomirova et al., 2020; Rose, Feldman, Jankowski, 2011; Rindermann, Neubauer, 2004). Despite some differences in these models, the fundamental importance of information processing speed was postulated as underlying higher-order cognition, in particular, intelligence or creativity (Rindermann, Neubauer, 2004; Fry, Hale, 2000). For example, a study with participation of Russian schoolchildren showed that the model that fit the data the best was one in which reaction time was a key predictor of intelligence, working memory, and number sense. These in turn contribute as a factor of individual differences to general academic success (Tikhomirova et al., 2020).

Studies have recorded statistically significant effects of sociocultural conditions on the structure of the relationships between cognitive abilities and the academic achievements of schoolchildren (Tucker-Drob, & Bates, 2016; DeNavas-Walt, Proctor, 2014; Schneeweis, Skirbekk, & Winter-Ebmer, 2014). In a meta-analysis of research data from 45 countries, cross-cultural differences were associated, first, with the effectiveness of the functioning of the national education system at the level of r = 0.25 at *p* < 0.001 and the socioeconomic status of states at the level of r = 0.16 at *p* < 0.001 (Brouwers, Van de Vijver, Van Hemert, 2009). It was shown that in a less heterogeneous more effective educational environment the contribution of cognitive abilities to the formation of individual differences in academic success increases (Tosto et al., 2019; Tucker-Drob, & Bates, 2016). Additionally, greater school subject orientation of the national education system, such as towards mathematics, can influence the educational achievements of schoolchildren in this subject (Paik et al., 2011). Finally, more intensive cognitive development under conditions of higher socioeconomic status has been reported to lead to changes in its relationship with academic success in different sociocultural environments (von Stumm & Plomin, 2015; Nisbett et al., 2012; Rindermann et al., 2010).

This cross-cultural study aims to identify relationships between cognitive abilities and academic success at the primary level of school education.

The analysis of the models of these relationships was conducted using samples of primary school age children from Russia and Kyrgyzstan, two countries that have a similar organization of the national education system but differ in the level of socioeconomic development and the effectiveness of education. In the 2020 International Ranking of the United Nations Development Program, which reflects achievements in the field of health care, education and social security, the Russian Federation is included in the group of countries with a very high level of human development (52 places), and the Kyrgyz Republic is included in the group of countries with an average level of human development (120 places). According the OECD’s Programme for International Student Assessment Kyrgyz 15-year-old schoolchildren had very low level performance in mathematics, with reading and science knowledge ranked at the bottom. Russian schoolchildren by contrast scored average.

It was concluded that these socioeconomic differences in the effectiveness of the functioning of national educational systems may lead to differences in the degree of development of cognitive processes, as measured by tests, but not in the relationship between these cognitive characteristics and learning success at the primary level of school education.

## Methods

### Participants

The study involved 732 schoolchildren aged 7.7 to 11.8 years studying in two public schools in Russia and Kyrgyzstan. These schools were similar according to the criteria of the departmental affiliation, qualifications and structural characteristics of the teaching staff, with corresponding the curricula in all school subjects, regional ratings, etc. These schools were state schools with no selection of pupils. In both samples, all children studying in the schools at Grades 2 to 4 participated in the study. The reasons for any child’s non-participation were limited to illness or absence from school on the date of testing.The instructional language in both schools was Russian.

The Russian sample included 355 pupils in grades 2–4, aged 7.8 to 11.7 years, of which 50.1% were girls. The average age is 9.84 years. All children spoke Russian. The Kyrgyz sample consisted of 377 pupils in grades 2–4, aged 7.7 to 11.8 years, of which 51.5% were girls. The average age is 9.75 years. Kyrgyz-speaking children accounted for 84.1%, and Russian-speaking children accounted for 15.9%.

All subjects gave their informed consent for inclusion before they participated in the study. Parental informed consent was obtained for all participants. The study was conducted in accordance with the Declaration of Helsinki, and the protocol was approved by the Ethics Committee of the Psychological Institute of the Russian Academy of Education (project identification code 2016/2–12). Data analysis was conducted using anonymized personal data.

### Procedure

At the end of the academic year, all study participants completed computerized test battery tasks aimed at assessing information processing speed, visuospatial working memory and number sense.

Additionally, participants completed the paper-and-pencil test assessing their nonverbal intelligence. Data collection was conducted in the computer science room strictly according to the developed protocol under the supervision of the researcher. Quarterly grades were recorded at the end of the school year with the consent of the participants and their parents.

### Measures: cognitive abilities

*Information processing speed* was assessed using a “Choice reaction time” test with four alternative choices (Tikhomirova et al., 2020). Numbers ranging from 1 to 4 appear 40 times on the computer screen in a random order at varying time intervals— from 1 to 3 seconds. The pupils need to press the key that corresponds to the number on the screen as quickly and as accurately as possible. The accuracy of the answer (correct/incorrect key pressed) and the reaction time are both recorded. In the present study, the reaction time indicator is used only for correct answers.

*Visuospatial working memory* was assessed using the “Sequences” test designed based on the “Corsi block-tapping” test (Tikhomirova et al., 2020). A certain number of cubes appear on the computer screen and “light up” one after the other in a certain sequence with an interval of 1 second. The minimum number of cubes in a sequence is 4, and the maximum is 9. The pupils are required to repeat the presented sequence by clicking on the cubes in the same order as they lit up using a computer mouse. The present study uses the indicator of the number of correctly reproduced sequences.

*Number sense*, which is associated with the perception and manipulation of non-symbolically expressed quantities, was assessed using the “Number sense” test (Kuzmina et al., 2020) in which an array of yellow and blue dots, differing in size and number, appears on the computer monitor within 400 ms. The tasks are grouped into three blocks of 50 arrays. The number of dots of each colour in the task varies from 5 to 21. The pupils need to decide within 8 seconds which colour dots— blue or yellow— there are more of, and press the corresponding colour key on the keyboard. The indicator of the total number of correct answers was used in the statistical analysis of the present study.

*Nonverbal intelligence* was measured using the printed version of the “Standard Progressive Matrices” test (Raven, 2003). The test consists of 60 tasks grouped into 5 series. The tasks become progressively more difficult within each series and from series to series. The pupils are required to select the missing element of the matrix from among 6 or 8 options. This study uses the indicator of the total number of correct answers on this test.

### Measures: Academic achievement

The academic success indicator was calculated on the basis of the quarterly grades of the primary school students in Russian language, mathematics and biology, as assessed by school teachers.

### Statistical analysis

During the first stage, the descriptive statistics of the cognitive abilities and academic success were calculated for the Russian and Kyrgyz samples of primary school children. One-way analysis of variance was conducted to understand cross-cultural differences in cognitive abilities— information processing speed, visuospatial working memory and nonverbal intelligence. Differences in all analysed indicators between Russian- and Kyrgyz-speaking schoolchildren studying in Kyrgyzstan were logged.

During the second stage, correlation analysis at the Russian and Kyrgyz samples was conducted to study the cross-cultural specifics of the relationship of cognitive abilities to academic success (IBM SPSS 20.0 statistical package).

During the third stage, theoretical models of the relationship between cognitive development and academic success were tested by applying the structural equation modelling method to the samples of Russian and Kyrgyz schoolchildren (OpenMX statistical package). The decision to accept or reject the tested model was made on the basis of the values of the conformity quality tests: root mean square error of approximation (RMSEA) <.06, the 95% confidence interval for RMSEA low = 0.00 and high <.08, comparative fit index (CFI) compliance score >.95, and the Tucker-Lewis index (TLI) > 0.90 (Hu, Bentler, 1999).

During the course of the structural modelling, the following theoretical models of the relationship between the cognitive development and academic success were tested on each of the analysed samples of schoolchildren.

Model 1: Cognitive indicators affect academic success through the latent variable of general cognitive ability ‘*g*’;

Model 2: The cognitive indicators— information processing speed, visuospatial working memory, number sense and nonverbal intelligence— contribute to the factor of general academic success ‘e’ (‘education’) allocated on the basis of grades in mathematics, Russian language and biology;

Model 3: Information processing speed is a key predictor of nonverbal intelligence, working memory and number sense, which in turn contribute to the ‘e’ factor of general academic success.

## Results

The statistical analysis included cognitive abilities— information processing speed, visuospatial working memory, number sense and nonverbal intelligence— and teachers’ assessments in Russian language, mathematics and biology as indicators of academic success.

*Table 1* shows mean and standard deviations (in brackets) for the analysed indicators in the groups of primary school age children from Russia and Kyrgyzstan.

**Table 1:** Descriptive statistics of the cognitive abilities and academic success

Indicator	Schoolchildren from Russia	Schoolchildren from Kyrgyzstan
Information processing speed	.99 (.27)	1.03 (.25)
Visuospatialworking memory	2.70 (1.84)	2.75 (1.84)
Number sense	94.21 (14.15)	96.03 (14.23)
Nonverbal intelligence	38.21 (9.5)	32.13 (10.60)
Grades in Math	4.02 (0.6)	4.20 (0.6)
Grades in Russian language	3.87 (0.6)	4.19 (0.7)
Grades in Biology	4.45 (0.5)	4.64 (0.5)

*Table 1* shows the total number of correctly completed test tasks for the visuospatial working memory, number sense and nonverbal intelligence. The minimum and maximum possible values are 0 and 12 for the “Sequence” test, 0 and 150 for the “Number sense” test, and 0 and 60 for the “Standard Progressive Matrices” test. Information processing speed is presented in seconds. The values of the quarterly grades in school subjects range from 2 to 5.

According to *Table 1*, for the visuospatial working memory and number sense indicators, slightly higher mean values were obtained by the group of Kyrgyz schoolchildren; and for information processing speed and nonverbal intelligence, higher values were obtained by the Russian schoolchildren.

Grades in all analysed subjects, including Russian language, were higher for Kyrgyz schoolchildren. It should be emphasized that the subjective nature of teachers’ assessments and differing criteria of success in teachers’ assessments of acquired knowledge in each of the national educational systems make it impossible to directly compare cross-cultural differences in pupils’ performance. During the course of further analysis, based on the grades in the three school disciplines, an indicator of overall academic success was calculated, which was applied in the context of its relationship with the cognitive sphere only within the context of a particular cultural group.

To assess the cross-country differences in and extent of the cognitive abilities, an analysis of variance was performed, and the following indicators were introduced as the dependent variables: information processing speed, visuospatial working memory, number sense, and nonverbal intelligence. Levene test values (*p* > 0.05) indicate the equality of the variances of all analysed cognitive variables for the samples compared.

*Table 2* shows the results of the one-way analysis of variance where the factor of the country of residence— Russia or Kyrgyzstan— was used as the categorical factor.

As per *Table 2*, statistically significant differences between groups of primary school age children from Russia and Kyrgyzstan were obtained in terms of information processing speed with a small effect size of 1% and nonverbal intelligence with an effect size of 8% (*p* < 0.001). For both indicators, higher results were obtained for the sample of Russian schoolchildren (see *Table 1* for the descriptive statistics). Visuospatial working memory and number sense did not statistically significantly differ in the groups of schoolchildren studying in Russia and Kyrgyzstan (*p*>0.05).

**Table 2:** The results of analysis of variance on cognitive abilities

Indicator	Sum of Squares (*SS*)	F-statistics (*F*)	*p*-value (*p*)	Effect size (2)
Information processing speed	0.79	11.31	0.00	0.01
Visuospatial working memory	91.59	21.44	0.10	0.001
Number sense	755.03	3.66	0.06	0.001
Nonverbal intelligence	9293.65	91.48	0.00	0.08

Analysis of variance was also conducted to assess the differences between Russian and Kyrgyz-speaking schoolchildren from Kyrgyzstan in terms of cognitive indicators and school grades. According to the results, no significant differences were found between schoolchildren that were native and non-native Russian speakers studying in Kyrgyzstan for all the analysed cognitive development indicators and teachers’ assessments (*p*>0.05).

### Correlation analysis

The relationship between cognitive abilities and academic achievement in Math, Language and Biology was studied during the course of the correlation analysis.

*Table 3* shows Spearman’s correlation coefficients between information processing speed; visuospatial working memory; number sense; nonverbal intelligence; and success in learning mathematics, Russian language and biology in Russian (top line) and Kyrgyz (bottom line) samples.

**Table 3:** Correlation matrix for the cognitive abilities and academic achievement

	IPS	VSWM	NS	NI	Lang	Math	Bio
VSWM	–0.34** –0.38**	1					
NS	–0.21** –0.21**	0.33** 0.28**	1				
NI	–0.26** –0.39**	0.42** 0.42**	0.37** 0.34**	1			
Lang	–0.02 –0.03	0.15** 0.15**	0.24** 0.20**	0.47** 0.22**	1		
Math	–0.09* –0.06	0.22** 0.21**	0.26** 0.22**	0.48** 0.27**	0.79** 0.74**	1	
Bio	–0.06 0.11	0.17** –0.06	0.22** 0.12*	0.43** –0.07	0.74** 0.52**	0.72** 0.49**	1

Note. IPS =information processing speed, VSWM = visuospatial working memory, NS = Number sense, NI = Nonverbal intelligence, Lang = Grades in Russian language, Math = Grades in Math, Bio = Grades in Biology. ** p < 0.01; * p < 0.05.

As shown in *Table 3*, in the relationship between cognitive development and school achievement, there are both similarities and differences in the samples of Russian and Kyrgyz schoolchildren.

In particular, in both cross-cultural samples, information processing speed was unrelated to success in learning Russian language and biology (*p*>0.05). Differences were obtained for mathematics. Only in Russian schoolchildren was a weak but statistically significant correlation found.

Visuospatial working memory and nonverbal intelligence were related to Russian language and mathematics assessments in both samples of children at the primary level of school education, and differences were obtained for biology. Only in the Russian sample was a statistically significant correlation found. As for the relationship between number sense and academic success for all analysed school subjects, both the Russian and Kyrgyz samples of schoolchildren were completely similar.

The structure of the relationships between the cognitive abilities— information processing speed, visuospatial working memory, number sense and nonverbal intelligence— is characterized by the similarity of the number and strength of relationships in the analysed samples of schoolchildren. Furthermore, in both sociocultural samples, the strongest relationship was obtained for nonverbal intelligence and visuospatial working memory (r = 0.42, *p* < 0.01), and the weakest relationship was obtained for information processing speed and number sense (r = 0.21, *p* < 0.01). In general, the coefficients of the correlation between the indicators of the cognitive sphere are moderately strong.

The relationships between the indicators of success in learning Russian, mathematics and biology in the sample of Russian schoolchildren were characterized by high correlation coefficients (0.72 < r < 0.79 at *p* < 0.01); and in Kyrgyz schoolchildren, these relationships were mostly moderate. An exception was the relationship between school grades in Russian language and mathematics. As in the Russian sample, the correlation coefficient in the sample of Kyrgyz schoolchildren reached a value of 0.74 at *p* < 0.01.

### Structural equation modeling

Three models of the relationship between the cognitive development and academic success were tested using the structural equation modelling method on samples of primary school age children from Russia and Kyrgyzstan.

According to Model 1, the cognitive indicators influence success in learning school subjects through the latent variable of general cognitive ability. Model 2 assumed the opposite influence of all the analysed cognitive abilities on general academic success calculated on the basis of school grades in mathematics, Russian language and biology. In Model 3, the baseline cognitive metric–information processing speed–is a key predictor of nonverbal intelligence, working memory, and number sense, which in turn contribute to individual differences in general academic success. The analysis of the structural models showed that the tested theoretical Models 1 and 2 corresponded poorly to the empirical data of Russian and Kyrgyz children at the primary level of general education (RMSEA>0.08, CFI< 0.95, TLI < 0.90, and X^2^significant (p < 0.05)). However, Model 3 best matched the data obtained in both the Russian and Kyrgyz samples.

The fit indices of theoretical Model 3 to the empirical data of samples of primary school age children from Russia and Kyrgyzstan are presented in *Table 4*.

**Table 4:** Fit indices of theoretical Model 3 to the empirical data of Russian and Kyrgyz samples

	AIC	BIC	CFI	TLI	RMSEA	RMSEA low	RMSEA high
Schoolchildren from Russia	6647.17	–14125.86	0.996	0.991	0.027	0.000	0.055
Schoolchildren from Kyrgyzstan	6515.67	–13495.08	0.942	0.894	0.059	0.004	0.024

*Note. AIC =Akaike information criterion, BIC = Bayesian information criterion, CFI= comparative fit index, TLI = Tucker Lewis index, RMSEA = root mean square error of approximation, RMSEA low = the lower limit of the 95% confidence interval for RMSEA, RMSEA high = upper limit of 95% confidence interval for RMSEA*

According to *Table 4*, the fit indices for Model 3 indicate good agreement with empirical data. Thus, for the sample of schoolchildren studying in Russia, RMSEA 0.06, 95% confidence intervals — RMSEA low = 0.00 and RMSEA high< 0.08, CFI> 0.95, and TLI> 0.90. Furthermore, the X^2^ value is not significant (*p* > 0.05), which reflects a good fit of the model. Satisfactory fit indices were obtained for the sample of schoolchildren from Kyrgyzstan (see *Table 4*), and the X^2^ value was not significant (*p* > 0.05).

**Figure 1.**
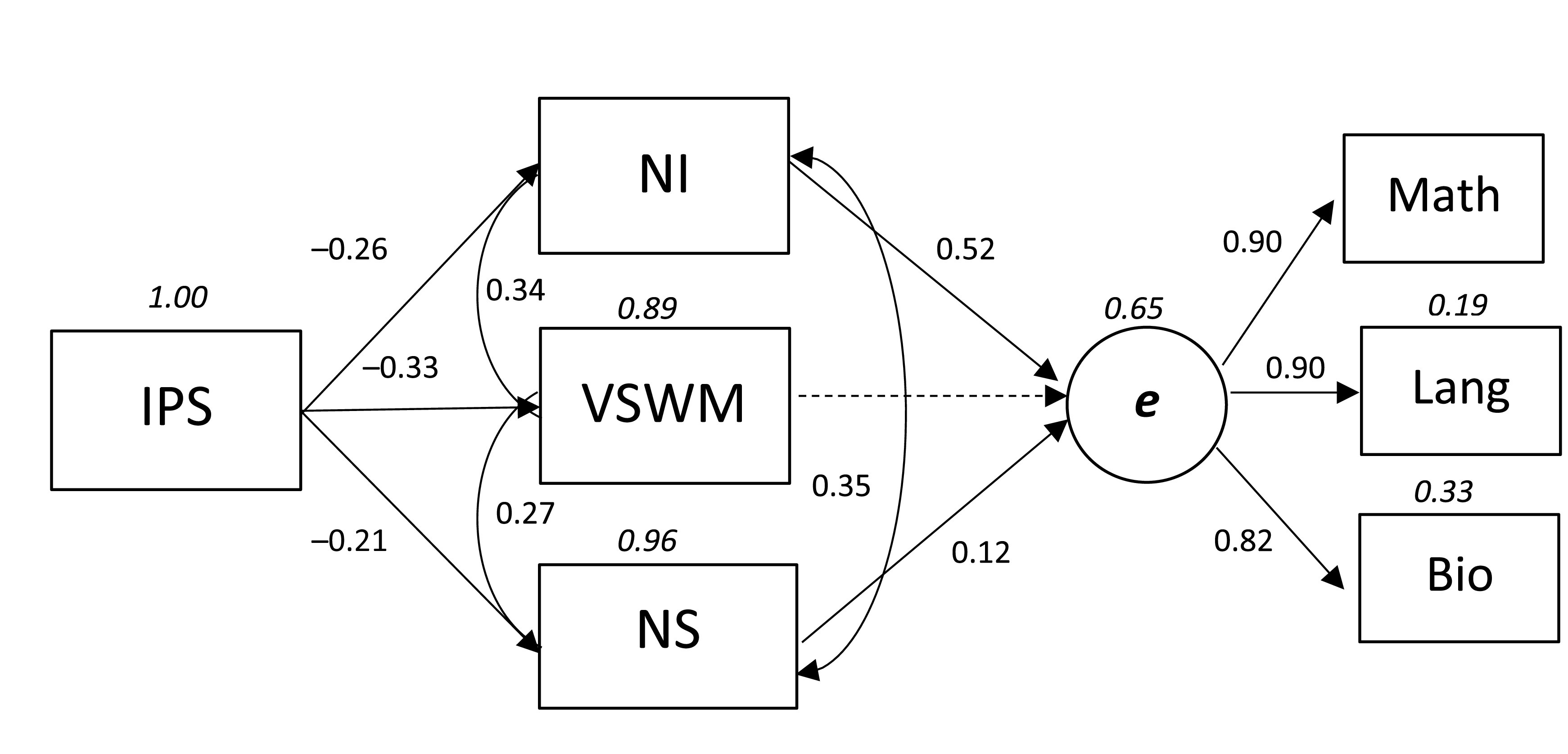
Model of the relationship between the cognitive abilities and academic achievement for the sample of Russian schoolchildren

*Figure 1* show a model of the relationship between information processing speed (IPS), visuospatial working memory (VSWM), number sense (NS), nonverbal intelligence (NI) and academic success for the sample of primary school children from Russia. The model included standardized structural coefficients (*p* < 0.05), and dashed lines were used to indicate statistically nonsignificant relationships (*p* > 0.05).

As seen in *Figure 1*, the model considers general academic success as a latent variable based on the three indicators of academic success: mathematics, Russian language, and biology performance. These indicators of academic success were almost equally loaded on the latent factor of general academic success ‘e’ (ranging from 0.82 to 0.90).

According to this model, information processing speed is considered the basic cognitive indicator underlying higher order cognitive abilities: intelligence, working memory and number sense. In turn, these cognitive abilities influence academic success. Comparing the standardized structural coefficients at the primary level of school education, information processing speed had the most influence on working memory (β = –0.33), followed by the nonverbal intelligence (β = –0.26) and number sense (β = –0.21) indicators. The regression weights between nonverbal intelligence, visuospatial working memory, and number sense ranged from 0.27 to0.35. Of all cognitive abilities, nonverbal intelligence had the greatest influence on the academic success factor (β = 0.52).

Consequently, in the sample of Russian schoolchildren, most of the influence of the cognitive sphere on academic success can be seen in the trajectory of the indirect influence of information processing speed through nonverbal intelligence. Standardized path coefficients, calculated in accordance with the principles of structural equation modelling (Rinderman, Neubauer, 2004; Hu&"Bentler, 1999), statistically confirm this fact. Th us, the regression weight of the path “Information processing speed— Nonverbal intelligence— Academic achievement” is –0.26 × 0.52 = –0.13. The standardized structural coefficients for other possible paths in which the cognitive development might influence academic success are shown below. It must be noted that a model with a direct influence of information processing speed on the academic success factor has unsatisfactory indicators of fit. According to *Figure 1*, the residual variance of general academic success is 0.65. Thus, in a sample of primary age schoolchildren from Russia, using the analysed cognitive abilities, 35% of the variance in academic achievement was explained.

**Figure 2.**
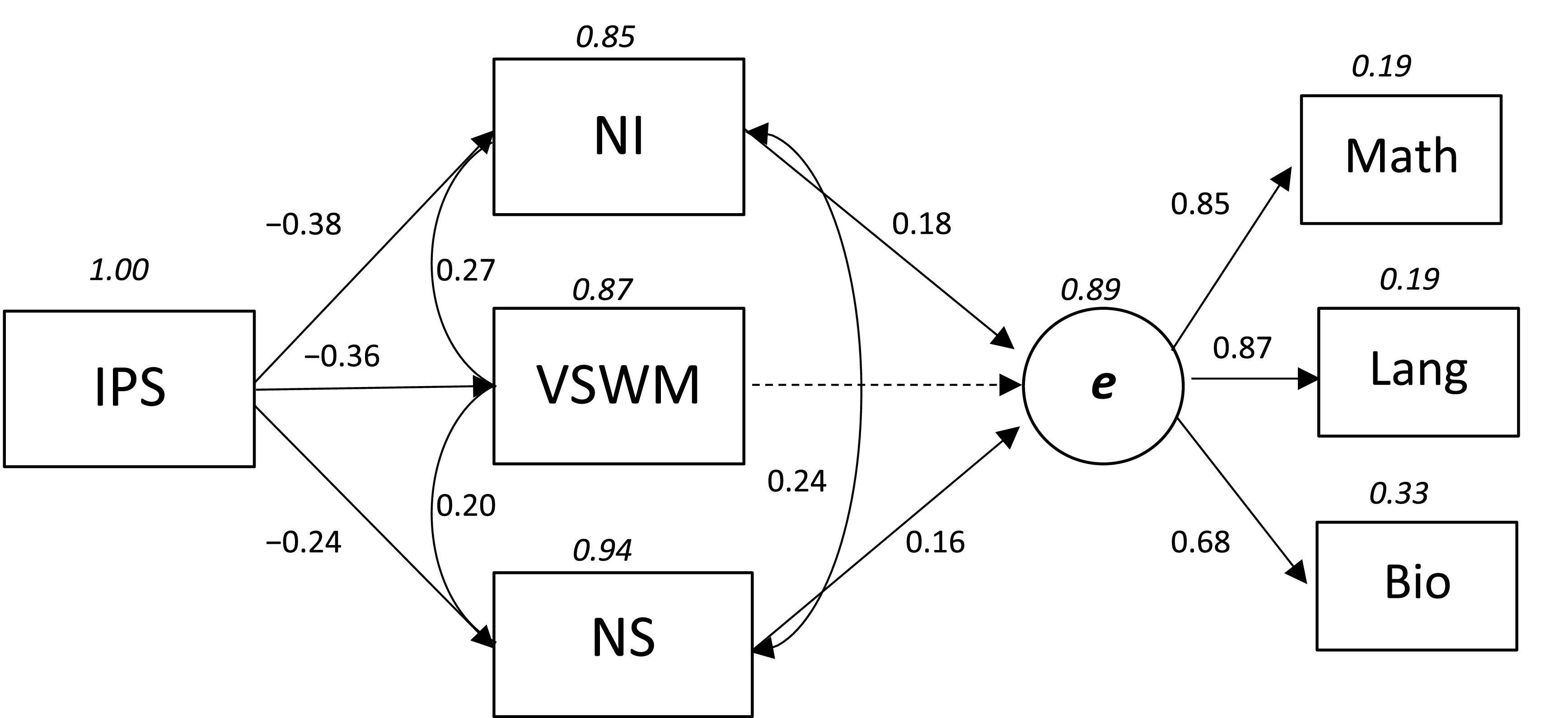
Model of the relationship between the cognitive abilities and academic achievement for the sample of Kyrgyz schoolchildren

*Figure 2* shows a model of the relationship between information processing speed (IPS), visuospatial working memory (VSWM), number sense (NS), nonverbal intelligence (NI) and academic success for the sample of schoolchildren from Kyrgyzstan. The model shows standardized structural coefficients (*p* < 0.05), and dashed lines are used to indicate statistically nonsignificant relationships (*p* > 0.05).

According to *Figure 2*, in the sample of children from Kyrgyzstan, school performance indicators in mathematics, Russian language and biology were loaded on the latent factor of general academic success to varying degrees (from 0.68 to 0.87).

Comparison of standardized structural coefficients confirmed the fact that information processing speed had the most influence on nonverbal intelligence (β = –0.38), followed by the working memory (β = –0.36) and number sense (β = –0.24) indicators. The regression weights between nonverbal intelligence, working memory, and number sense ranged from 0.20 to 0.27. As in the sample of Russian primary schoolchildren, of all the cognitive abilities, nonverbal intelligence had the greatest influence on general academic success at the primary school education but with a significantly lower contribution (β = 0.18).

Structural equation modelling yielded standardized path coefficients that confirmed that in the Kyrgyz sample, the way cognitive functioning influences academic success is the indirect impact of information processing speed through nonverbal intelligence, as in the Russian sample. The regression weight of the path “Information processing speed — Nonverbal intelligence — Academic achievement” is–0.38 × 0.18 = –0.07. According to *Figure 2*, in the Kyrgyz sample, the residual variance of general academic success is0.89. This means that in the sample of primary school age children from Kyrgyzstan, only 11% of the variance in academic achievement was explained using the analysed cognitive abilities.

## Discussion

During the course of this study, a cross-cultural analysis of the structure of the relationships between the cognitive abilities and academic achievement was conducted using groups of primary school children studying in countries with different socioeconomic statuses and educational effectivenesses— Russia and Kyrgyzstan.

Among the cognitive development indicators, cross-cultural differences in nonverbal intelligence were observed with an effect size of 8%. According to the results, primary-level schoolchildren studying in Kyrgyzstan performed worse on the “Standard Progressive Matrices” test than their Russian peers. This result concords with the data of cross-cultural studies on the “sensitivity” of intelligence measured by the “Standard Progressive Matrices” test to educational conditions (von Stumm & Plomin, 2015; Nisbett et al., 2012; Rindermann et al., 2010). Thus, studies, including those with the participation of Russian and Kyrgyz schoolchildren, reported advantages of children studying in more favourable macro and micro socioeconomic conditions (Kuzmuna et al., 2020; Nisbett et al., 2012). Additionally, it is noted that cross-country differences are reduced during the course of schooling (Kuzmuna et al., 2020).It has also been shown that schooling leads to a gradual reduction in the range of variability in a number of cognitive abilities, such as intelligence, information processing speed, and number sense associated with the ability to accurately determine the position of a number on a number line (Tikhomirova et al., 2020; Nisbett et al., 2012). Th us, a longitudinal study reports a significant decrease in interindividual di# erences in terms of the accuracy of assessing symbolically expressed quantities under the influence of formal schooling from the first to the fourth year (Kuzmina et al., 2020).

In the present study, no differences were found between schoolchildren from Kyrgyzstan with Russian and Kyrgyz as their native languages, which may also confirm the effects of the influence of the socioeconomic status of the country overall. Minor cross-cultural differences were obtained for the information processing rate with an effect size of 1%. Regarding visuospatial working memory and number sense, no differences were found between younger schoolchildren studying in Russia and Kyrgyzstan. Similar data were obtained in studies with the participation of respondents from other age and cultural groups (for example, Brown et al., 2012).

In the relationship between the cognitive abilities and success in learning various school subjects at the primary school education, both similarities and differences between schoolchildren studying in Russia and Kyrgyzstan were found.

Regarding the similarities, it should be noted that the relationships between visuospatial working memory and number sense and the indicators of successful learning of Russian language and mathematics are almost identical in strength. Additionally, in both samples, the connection between information processing speed and school achievement indicators was absent. An exception was a weak relationship between information processing speed and mathematics success in the sample of Russian schoolchildren. These data agreed with the results of studies conducted with the participation of Russian respondents (for example, Tikhomirova et al., 2020) and may hint towards the presence of indirect relationships with academic success. Despite the identified similarities in the relationship between the cognitive sphere and school performance, the analysis revealed some cross-cultural differences. For example, the most significant difference is the ratio of nonverbal intelligence and assessments in all analysed school subjects. In particular, it was shown that in the Russian sample of children at the primary level of school education, the strength of the relationship was almost twice as high when compared with that of the Kyrgyz sample. This result confirms assumptions about the greater role of cognitive abilities (in particular, intelligence) in the formation of individual differences in learning in favourable and homogenous educational environments (Tucker-Drob, & Bates, 2016).

The best indicators of fit with the empirical data for both samples of schoolchildren were found for the model with information processing speed as the basic predictor of intelligence, working memory and number sense, which then together contribute to general academic success. Nonverbal intelligence plays a central role in this model, and its importance for academic achievement has been repeatedly reported in studies with differing cultural and age contexts (Deary, Johnson, 2010; Ritchie, Bates, Deary, 2015). These data contribute to the notion of universal applicability of this model, already confirmed by previous studies, including those with the participation of schoolchildren from Russia (Tikhomirova et al., 2020). It should be noted that in the study with the participation of German schoolchildren, the most satisfactory model was recognized as that wherein information processing speed affects school achievements through higher order cognitive abilities such as intelligence and creativity only indirectly (Rindermann, Neubauer, 2004).

Along with the invariance of the model, the cross-cultural specificity of the relationships within this structure was shown. In particular, in the sample of Russian schoolchildren, the regression weight of the relationship between information processing speed and academic success through intelligence turns out to be more significant compared to the data of Kyrgyz schoolchildren (modulo0.13 versus0.07). Notably, the significantly greater contribution of intelligence to the indicator of general academic success in the Russian sample of primary school age children compared to the Kyrgyz sample is consistent with research data on the effects of national education systems on the structure of relationships between the cognitive sphere of schoolchildren and their educational achievements (Nisbett et al., 2012).

According to the results of this study, at the primary level of education, the contribution of the cognitive abilities to the general academic success of schoolchildren from Russia was estimated at 35% of the variance of the school achievement indicator; and in the sample of schoolchildren from Kyrgyzstan, the contribution was only 11%. In other words, in a more unified and effective educational environment (in terms of international rankings and assessments of student educational achievement), there was an increase in the role of cognitive indicators in individual differences in academic success at the initial level of general education. Such a tendency towards an increase in the influence of cognitive development on the success of schooling may be associated with the specifics of the requirements of developing educational programs and assessment of academic achievements in different sociocultural conditions.

## Conclusion

The cross-cultural analysis of the models of the relationship between cognitive development and academic achievement revealed a universal applicability for one of the models tested for primary-level schoolchildren studying in Russia and Kyrgyzstan. According to the structural equation modelling results, the baseline cognitive metric (information processing speed) was a key predictor of nonverbal intelligence, visuospatial working memory and number sense. These which together seem to contribute to individual differences in general academic success, and constitute a model best suited to the empirical evidence.

Along with the universality of the model, cross-cultural differences in the relationship between the cognitive development and school success indicators were revealed, differences yielding a significant decrease in the influence of the cognitive abilities of a schoolchild on his or her academic achievements, given less favourable educational conditions. Presumably, in such a case, other personal and/or motivational resources may make more significant contributions to the formation of individual differences in academic success as measured by teachers’ assessments.These results of our study can be used, we suggest, in educational practice to improve the efficiency of the functioning of the national education system.

Future research directions relate to the analysis of the joint influence of cognitive, personal and motivational traits on school achievement.

## Limitations

In this study academic achievements were measured only by grades in Russian language, mathematics and biology as assessed by school teachers.At the same time, various indicators— grades, standardized test assignments and state exams scores in school subjects can be used to assess school achievements.
